# Technical challenges of providing record linkage services for research

**DOI:** 10.1186/1472-6947-14-23

**Published:** 2014-03-31

**Authors:** James H Boyd, Sean M Randall, Anna M Ferrante, Jacqueline K Bauer, Adrian P Brown, James B Semmens

**Affiliations:** 1 Centre for Data Linkage, Curtin University, Perth, Western Australia; 2 The Birchman Group, Perth, Western Australia

**Keywords:** Medical record linkage, Automatic data processing, Medical informatics computing

## Abstract

**Background:**

Record linkage techniques are widely used to enable health researchers to gain event based longitudinal information for entire populations. The task of record linkage is increasingly being undertaken by specialised linkage units (SLUs). In addition to the complexity of undertaking probabilistic record linkage, these units face additional technical challenges in providing record linkage ‘as a service’ for research. The extent of this functionality, and approaches to solving these issues, has had little focus in the record linkage literature. Few, if any, of the record linkage packages or systems currently used by SLUs include the full range of functions required.

**Methods:**

This paper identifies and discusses some of the functions that are required or undertaken by SLUs in the provision of record linkage services. These include managing routine, on-going linkage; storing and handling changing data; handling different linkage scenarios; accommodating ever increasing datasets. Automated linkage processes are one way of ensuring consistency of results and scalability of service.

**Results:**

Alternative solutions to some of these challenges are presented. By maintaining a full history of links, and storing pairwise information, many of the challenges around handling ‘open’ records, and providing automated managed extractions are solved. A number of these solutions were implemented as part of the development of the National Linkage System (NLS) by the Centre for Data Linkage (part of the Population Health Research Network) in Australia.

**Conclusions:**

The demand for, and complexity of, linkage services is growing. This presents as a challenge to SLUs as they seek to service the varying needs of dozens of research projects annually. Linkage units need to be both flexible and scalable to meet this demand. It is hoped the solutions presented here can help mitigate these difficulties.

## Background

Record linkage is the process of bringing together data relating to the same individual from within and between different datasets. When a unique person based identifier exists, this can be achieved by simply merging datasets on the identifier. When this identifier does not exist, some form of data matching or record linkage is required. Often, statistical or probabilistic matching processes are applied to records containing personally identifying information such as name and address.

Record linkage techniques are widely used in public health to enable researchers to gain event based longitudinal information for entire populations. In Australia, research carried out using linked health data has led to numerous health policy changes [1,2]. The success of linkage-based research has led to the development of significant national linkage infrastructure [3]. Comparable record linkage infrastructure exists in few other countries (e.g. England [4], Wales [5], Canada [6], Scotland [7]). The demand for linkage services to support health research, as well as for other forms of human and social research, is increasing [8-10].

There are differing operational models for the provision of record linkage services; however, some elements of the current infrastructure are similar. For example, in Australia and Wales, record linkage is conducted by trusted third parties or specialised linkage units (SLUs). SLUs are usually located external to the data custodians and researchers. This provides an element of separation, which enhances privacy protection [11]. Using specific software, including where appropriate privacy preserving record linkage techniques [12], SLUs engage in high quality data matching. Linkage results (keys) are either returned to the data custodian or forwarded directly to the researcher (depending on the model in use). Once de-identified data has been merged using the linkage keys, analysis of linked data can occur.

The record linkage processes used by SLUs can be quite complex and involve many components e.g. data cleaning and standardisation, deterministic and/or probabilistic linkage, clerical review, etc. Many factors influence the consistency and quality of linkage results [13].

Notwithstanding the complexity of record linkage, SLUs face additional technical challenges in providing linkage ‘as a service’ for research. The extent of this functionality, and approaches to solving these issues, has had little focus in record linkage literature. Few, if any, of the record linkage packages or systems in use by SLUs today include the full range of functions required of/by these entities.

The purpose of this paper to identify and discuss some of the technical issues associated with the provision of record linkage services, and to propose solutions to these problems. Of particular interest is the array of challenges associated with on-going linkage (i.e. continuous linkage of changing datasets over time). These issues have not been previously addressed in the literature, and it is the aim of this paper to do so.

## Methods

The role of SLUs has become more prominent in the research infrastructure landscape and the level and complexity of demands placed on them for linkage services has increased. While there are a variety of techniques available to undertake record linkage such as deterministic rules-based methods, sort and match algorithms [14], and probabilistic techniques [15,16], the tendency for most SLUs has been to implement a probabilistic framework, owing to its robustness, adaptability (particularly in relation to linkage of large datasets – see, for example Clark and Hahn [17]) and high-quality output [18,19]. Probabilistic methods involve sophisticated blocking techniques (to streamline comparisons) and the application of matching methods that incorporate both deterministic and probabilistic comparisons [20-22]. In recent times, there has been extensive work on extending probabilistic approaches and improving efficiency using advances in technology [23,24]. However, beyond the complexity of the linkage process *per se*, there are other technical challenges that present to SLUs. These include the general management of data, handling different linkage scenarios, the management of routine, on-going linkage (and the complexity of storing and handling changing data), the need for automation and the ever present need to accommodate larger sized datasets. In this section we discuss each of these emerging problems.

### General management of data

As the number of linkage projects increase, SLUs need robust, efficient methods of managing all forms of data. These include: incoming data from custodians (which need to be maintained in a secure environment, owing to identifying data items and which need to be cleaned and standardised [25] before being used in record linkage); outgoing data (i.e. the linkage keys that are subsequently delivered to others); detailed information about record linkage processes themselves and key decision factors (i.e. linkage strategies, weights, threshold settings, clerical review decisions); linkage results (matched pairs and group membership); and any other value-added information (e.g. geocoding information for addresses).

To ensure robust and reliable linkage operations, the SLUs require close integration between the record linkage software and enterprise level databases. This will help the management of the information resources as the volume of linked data increases.

### Handling different linkage scenarios

The linkage requirements of research projects vary. Some research projects require a ‘simple’ once-off linkage of one or more existing datasets, while others require more intricate linkage of datasets (e.g. genealogical linkage). SLUs need the ability to handle various linkage scenarios including both project based (create and destroy) and ongoing linkage research projects.

**‘Project based linkage’** is arguably the simplest scenario. This is where one or more datasets are required to be linked together for a single research project. These datasets are to be linked to each other, with the links only to be used for a specified research project. Based on the data agreements for the project; the datasets, and the links, often require to be deleted/destroyed after the project has completed.

**On-going linkage.** As systems, processes and relationships mature, SLUs typically move from a ‘project’ based approach, where data is linked for each specific research project and then the links are discarded when no longer required, to an on-going approach, where a central core of links is created and maintained over time and re-used for multiple research projects. As new records are added to the system, the links are updated. This approach dramatically reduces effort and improves linkage quality, as the same data are not required to be re-linked over and over with the impact of quality intervention and clerical review is not lost [26]; however, this introduces additional challenges in terms of the volume, speed and quality of matches and the management of associated linkage keys over time is itself complex.

Despite the vast array of record linkage software packages available, most focus on linking files on a ‘project’ basis, that is, linking a single file to itself (internal linkage) or linking two files to each other at a single instance in time. Currently there are a range of desktop applications that perform this function and although these are usually easy to implement and use, they can struggle to handle medium (>1 million) and large scale (>10 million) linkages [27]. Few, if any, commercial packages exist which have the capacity and functionality to undertake on-going record linkage. As a consequence, these complexities have been resolved in ad hoc ways by individual linkage units.

Alternative approaches to on-going incremental linkage have been developed in recent years, including those outlined by Kendrick [21,28] in his description of Best-link matching. Kendrick’s paper expands on the principles outlined by Newcombe [29,30] which describes the factors which could have an effect on the linkage quality, including the likelihood that a record in one file is represented in the matching file.

**Other linkage scenarios.** There are occasional scenarios where on-going linkage may not be possible, or the most appropriate solution. A SLU needs to understand requirements in both the long and short term, and how it can accommodate both ‘project based’ and ‘on-going’ linkage requests, if at all.

Another linkage scenario often dealt with by SLUs is ‘*bring your own’* linkage. This is where a researcher who has collected information on a study cohort wishes to link this data to another dataset which may or may not already exist in the linkage system. While this researcher’s data should link to the required dataset(s), there is no requirement that it should form part of the on-going system.

### Challenges associated with on-going linkage

There are several considerations that need to be addressed before implementing an on-going linkage system; these issues typically do not appear in simpler, project based linkage operations. These differences are subtle and are mainly a result of the intricacies of managing data over time. Each of the approaches has their strengths and weaknesses and their applicability or suitability will depend on project requirements.

**Figure 1:**
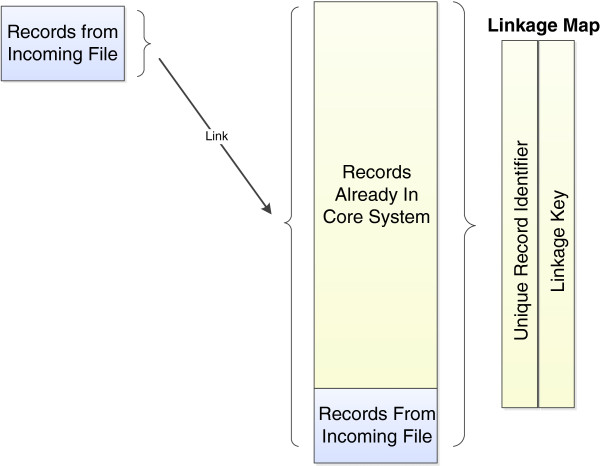
On-going linkage.

On-going linkage refers to the process of undertaking *routine, continuous linkage of (changing) datasets over time*. In on-going linkage, previously created links are retained by the system, and added to on the arrival of new records from the same datasets. New records entering the system needed to link to other new records (i.e. internally linked) as well as to existing records that are currently in the system (see Figure 1).

### *On-going linkage and the management of ‘open’ record*s

In project based record linkage, a linkage unit is typically supplied with a series of complete or ‘closed’ datasets which are required for a research project. These are then linked at a single point in time and the results given to the researcher. In on-going linkage, the necessary datasets are provided to units on a routine and, often, incremental basis. For example, a dataset may be supplied on a monthly basis. This dataset would contain new records for that month, as well as records that were updated during that month. Record received in one month may be amended, or completely removed from the dataset in the next month. An approach to handling new, amended and deleted records is required for on-going linkage.

In order to ensure the integrity of the linkage map and to avoid a re-link of all records, the linkage system should have the ability to detect and handle records which have been amended. This includes records which have had their personal identifying information changed (as these field values may influence matching decisions in earlier iterations of record linkage).

Similarly, the linkage system should have the ability to remove a record from the map. Ideally, this should occur in a way that removes any associations that may have been created by the existence of this record in the system.

### Maintaining a linkage map

On-going linkage systems require the maintenance of a central linkage map (a list of each record and the group they belong to). As linkage processes are continuous, the map needs to reflect results *as they occur over time* and for all records in the system, including those that are added or updated on an incremental basis.

### Accessing linkage map history

Maintaining a linkage map and its history has utility for researchers, as well as for SLUs. Once researchers receive their data, they may have queries relating to how specific records were linked together. The linkage map is constantly being updated as new records arrive, and as the linkage map may no longer contain these records/links, it may be unclear how these records were brought together. The same problem can occur when a researcher requests a second extraction of their data, (for instance, to receive additional records or content variables). When they receive their second extraction data, they find that the linkage map has changed (as new records have been added or quality fixes have been made) making it difficult to reconcile individual patient histories. For on-going linkage systems, a linkage unit must understand how it will accommodate project requests over time.

### Linkage automation

The main goal of adopting on-going linkage is to reduce the amount of time and effort required in conducting a large amount of project linkages, which are routinely re-linking the same data. Taking steps to automate parts of the linkage process fits in naturally with the aim of reducing operator time and effort and increasing scalability.

As on-going linkage systems typically contain a central linkage map which is used in every current and future linkage, the cost of an operator mistake can be very high. Systematic automation and reporting can be useful to ensure and control the quality of linkages over time.

## Results

A SLU may employ one of a number of models to ensure that linkage is carried out efficiently and securely while satisfying the linkage needs of the research. Some approaches to automation, linkage scenarios and the creation, management and use of a linkage map are presented below.

### Linkage automation

**Figure 2:**
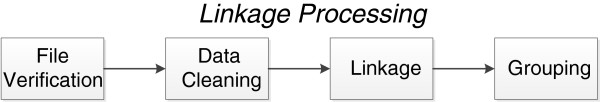
Steps in the linkage process.

Linkage processes are made up of several discrete steps (as shown in Figure 2), any number of which could be automated. At one end of the spectrum, the grouping process could be automated, with all other processes handled by operators. Upon verifying a file is correct, the operators clean the data and then link the file. When they are satisfied with linkage results, the linkage output is grouped into the linkage map.

Any system containing automation will require a process to ensure tasks are performed in an orderly manner. Looking at the sequence described in Figure 2, for example, a system could be implemented which examined a file to verify it contains the information it was expecting, before cleaning it in a predetermined way, and then linking the file in some predetermined or configurable way. The linkage results could then be added to the linkage map. A fully automated version of such a system would help fulfil the ‘linkage as a service’ model for some SLUs. Linkage services could be further extended so that data providers could connect to a portal to transmit a dataset, which is then automatically linked, with results automatically returned.

There are advantages and disadvantages to automated models of linkage service delivery. Using a fixed approach to cleaning and linking datasets ensures integrity and transparency, and where operators are routinely applying fixed approaches, these could also be added to automated processes. On the other hand, depending on the quality of the data, bespoke approaches to working with individual datasets may improve linkage quality over a one-size-fits-all approach.

### Linkage scenarios

Several options exist for handling the different likely linkage scenario requirements. One simple option is to use different linkage systems for different types of linkage scenarios. A SLU may choose to use one set of processes for project-based projects (only), while using an entirely different set of processes/tools for core, on-going linkage. The processes for project linkage may even include manual components.

A more complicated option is to design a single system for all linkage projects but which accommodates differing linkage scenarios for each specific project. Under this option, a linkage project may be configured to be on-going. The associated linkage map would also be ‘on-going’. A linkage project may also be designated to be a hybrid of projects and on-going linkage, that is, a linkage in which new project datasets are linked to records drawn from an existing, on-going datasets. Linkage results from these project, may, or may not, be added to the on-going linkage map, depending on the requirements of the research project and the likely quality of results.

The most appropriate option will depend, in part, on the number of different linkage scenarios facing SLUs. If requests for separate linkages and linkages to researcher datasets are common, then the first (simpler) option will require a large amount of operator time and resources, defeating the purpose of moving to on-going linkage, while the second may require a large amount of computational resources which may not be feasible.

### On-going Linkage

There are several possible methods for conducting on-going linkage and the linkage output will be influenced by a number of factors. One factor is the overlap of people between the files being matched i.e. how many new records have true matches in the existing linked file. Another influence is the size of the existing file, the larger the number of records involved in a probabilistic linkage the greater the likelihood that information will agree ‘by chance’ across records being compared.

These factors have an influence on the number of records brought together for linkage, the matching strategy and in the post-linkage processes that convert pairs of matched records into groups of records that are stored in a linkage map.

The relationship within and between files and the level of confidence in existing links/relationships are important considerations in the design and optimisation of linkage strategies.

For example, one approach is to link *all* records in the incoming dataset to *all* other records in the system. This method allows pairs to be created describing the relationships between *all* records in the system. With this approach, there are no expectations or assumptions made about how records match against each other or how they group together to become ‘sets’ of records that belong to the same individual. In terms of linkage strategy, this scenario represents a relatively unconstrained many-to-many linkage. If, however, the linkage task involves linking records to an authoritative record type (i.e. where only one high-quality record per person is known and maintained), then a one-to-one or many-to-one linkage may be more appropriate and there is opportunity to adapt matching strategies to leverage this knowledge [29,30].

**Figure 3:**
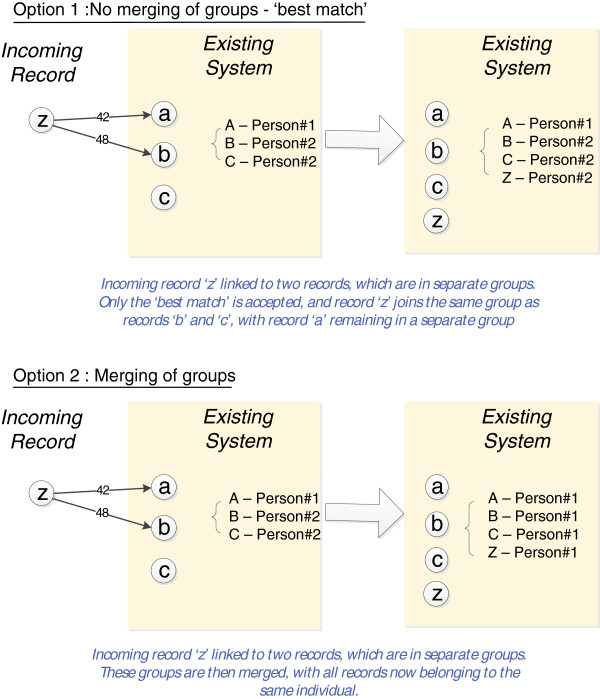
Methods for on-going linkage.

A related issue is whether or not to allow merging of groups in the linkage map. A linkage method known as ‘best-link matching’ [21] makes use of a population spine, which is a set of records already in the system that covers most of the population, and has been linked to a high standard. In this method, incoming records are unable to join together two groups already existing in the system– instead the ‘best link’ is chosen, and the incoming record is added to this group (Figure 3, Option 1).

This method uses underlying knowledge of the quality of the population spine to make decisions about future linkage results. Most SLUs accept that a small percentage of matches will be incorrect. In the situation where one of these matches merges two groups, the error is compounded and all records within these two groups are now incorrectly linked together^a^.

An alternate approach is to allow the merging of groups to occur. This method does not rely on the existence of a high quality reference dataset (spine). For this reason this method may be useful in a much greater range of circumstances.

There is an additional advantage to choosing strategies which allow merging of groups and which use all records in linkage. The advantage of this approach (and this approach only), is that the order of the incoming records does not affect system groupings. It is intuitive that this should be the case, as in practice the order of received records is typically highly dependent on contractual arrangements and other arbitrary preparations, which should not have an effect on the groups made by the system.

### Managing and accessing a changing linkage map

In on-going linkage, the linkage map is constantly changing and there may be requests from researchers to access results from previous linkages. There are several ways in which a SLUs can manage changing linkage maps and accommodate requests for past information. One solution is to take snapshots of the linkage system at the point of extraction for all research projects. This allows researchers access to the data and linkage map at the time of extraction and will solve the majority of the researchers queries, although the system would not be able to determine exactly why things have changed. While multiple snapshots of the system would take up a large amount of space, these do not necessarily need to be stored on on-going infrastructure, and could be moved elsewhere until required.

**Figure 4:**
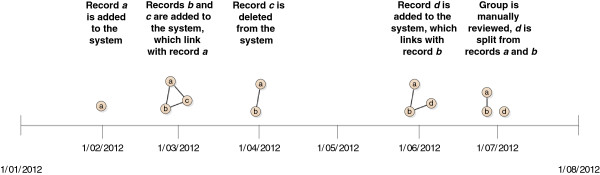
The full history of stored groups including the reasons for changes.

An alternative solution is to have a linkage map which stores the full history of groups, recording details of when additional records entered or left specific groups. This allows full understanding of how groups of records came together, as well as giving the ability to ‘roll-back’ to a point in time when an extraction for a researcher occurred (see Figure 4). Storing the full history of groups will likely take up more space in the linkage map; however, it provides greater flexibility in the extraction process and changes to groups are fully documented.

### Managing deleted, amended and ‘open’ records

#### Deleted records

**Figure 5:**
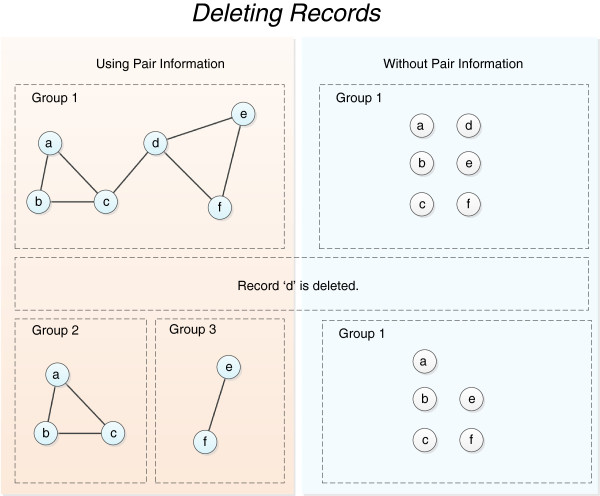
Methods for deleting records.

One option for managing deleted records is simply to remove them from the groups they are currently part of. The danger with this method is that the deleted record may have erroneously brought together two groups of records, which may now stay together indefinitely. A better approach is to unwind these groups by utilising the matching pair information used in creating these groups to discover how these groups would have looked had this deleted record not entered the system (Figure 5).

#### Amended records

There are several options available to manage amended records. One option is simply to amend the details stored in the database, without changing the system groupings. However these amended details may mean this record should belong to a different group, and that these links are actually in error.

An alternative option is to treat the amended record as a new record. In order to ensure the integrity of the linkage map, one must also identify and re-link any records that previously match to the record. This will ensure the new version is linked to the appropriate records.

By using pair information during deletion, and re-linking amended records, we can ensure the linkage map looks the same as if the deleted records and previous version of the amended records had never entered the system.

#### ‘Open’ records

Linkage systems that can handle deleted and amended records are better placed to accommodate the linkage of ‘open’ records. ‘Open’ records are those records where creation and end times vary and where the content of data may change between those dates. Many data providers only work with ‘closed’ records, which they can guarantee will not change. This process involves extensive validation and cleaning of the data before the file can be closed. This process is time consuming but ensures no changes to the linkage map once the file has been added. Some collection systems have ‘open’ records which can be amended over time. The advantage of ‘open’ files is that they can be updated to reflect amendments to records or deletions.

## Discussion

SLUs must service a range of record linkage needs from the research community. They must be able to deal with a range of linkage scenarios, from (simple) project linkage based approaches to complex on-going linkage. On-going linkage requires consideration of a number of additional time-sensitive issues which do not affect project based linkages. Despite the complexity, the advantages of moving to a more automated, efficient and sustainable way of conducting linkage far outweigh the intricacies of doing so. Table 1 summarises these key operational features of a linkage system and options available.

**Table 1:** Summary of issues and options for on-going linkage

**Operational feature**	**Options**
On-going linkage	- Link to most recent record in group vs. link to all records
	- Best-link matching vs. merging groups
Linkage automation	- Spectrum from fully automated to only the grouping process automated
Links stored	- No history stored
	- Snapshots stored
	- Full history stored within linkage map
Handling different linkage scenarios	- Only on-going linkage
	- Manual processes for project based linkage
	- Access to on-going linkage system used for project based linkage
	- Build system which can handle multiple scenarios
Amended and deleted records	- No handling of amended and deleted records
	- Amended records: Changing personal identifiers only vs deleting and re-linking
	- Deleted records: Simple removal, or using pair information to reconstitute groups

Several themes run throughout the issues presented in this paper. One is the trade-off between automation and bespoke approaches. Bespoke approaches will always be more flexible, but will always suffer from issues of transparency, maintainability and replicability. A second theme is the focus on issues and processes that complement and support the specialised activities of record linkage units. As presented in this paper, there are a number of key technical issues which must be understood and overcome in order for SLUs to deliver efficient record linkage ‘services’ for researchers.

There are several areas of further research required. To our knowledge, none of the options presented in this paper have been empirically compared against each other. However the employment of one option over another depends (typically) on assumptions about linkage quality, a measurable trait. If empirical research investigated the effect on linkage quality of several of these options over time given different datasets and other parameters, linkage units would be better equipped to decide on the most appropriate option for their systems.

A second area of research is related to the benefit of bespoke processes over automated processes. While it is assumed that automatic processes will likely produce lower quality results, the actual degradation in quality is not known. Research which tests and quantifies these effects is warranted. Until we know the true effect that automation has on linkage quality (if any), linkage units cannot make an informed decision about the benefit of this move.

## Conclusion

The process of conducting numerous linkages on a large scale is both complex and resource intensive. Linkage systems need to be both flexible and scalable to meet the future demands of enterprise-level record linkage. It is hoped the solutions presented here help reduce these difficulties.

## Endnote

^a^In this method false negatives found in the originating dataset used for the population spine will never be brought together no matter what additional information is found in other datasets. Additional records can provide new information which makes it clear that two records previously existing within the system actually belong to the same person. In these situations, ‘best-link matching’ will not be able to use this information to improve quality.

## Competing interests

The authors declare that they have no competing interests.

## Authors' contributions

Initial design and conception provided by JHB, AF and JS. Further technical design provided by AB, JKB and SR. First draft of manuscript provided by SR; subsequently edited significantly by JHB, AF and JS. All authors read and approved the final manuscript.

## Pre-publication history

The pre-publication history for this paper can be accessed here:

http://www.biomedcentral.com/1472-6947/14/23/prepub
